# The RNA Chaperone Hfq Is Important for Growth and Stress Tolerance in *Francisella novicida*

**DOI:** 10.1371/journal.pone.0019797

**Published:** 2011-05-05

**Authors:** Jacob R. Chambers, Kelly S. Bender

**Affiliations:** Department of Microbiology, Southern Illinois University, Carbondale, Illinois, United States of America; Charité-University Medicine Berlin, Germany

## Abstract

The RNA-binding protein Hfq is recognized as an important regulatory factor in a variety of cellular processes, including stress resistance and pathogenesis. Hfq has been shown in several bacteria to interact with small regulatory RNAs and act as a post-transcriptional regulator of mRNA stability and translation. Here we examined the impact of Hfq on growth, stress tolerance, and gene expression in the intracellular pathogen *Francisella novicida*. We present evidence of Hfq involvement in the ability of *F. novicida* to tolerate several cellular stresses, including heat-shock and oxidative stresses, and alterations in *hfq* gene expression under these conditions. Furthermore, expression of numerous genes, including several associated with virulence, is altered in a *hfq* mutant strain suggesting they are regulated directly or indirectly by Hfq. Strikingly, we observed a delayed entry into stationary phase and increased biofilm formation in the *hfq* mutant. Together, these data demonstrate a critical role for Hfq in *F. novicida* growth and survival.

## Introduction

*Francisella tularensis* is a gram-negative facultative intracellular pathogen and the etiologic agent of tularemia. Inhalation of as few as 10 organisms can result in development of pneumonic tularemia, a form of the disease associated with the highest morbidity and mortality. Due to its high infectivity and relative ease of dissemination, *F. tularensis* has been designated a category A select agent by the Centers for Disease Control and Prevention [1]. In humans, the most common form of tularemia is an ulceroglandular disease resulting from the bite of a bacteria-carrying arthropod vector or exposure to the infected blood of an animal through an abrasion in the skin [2]. Numerous arthropod vectors have been implicated in the transmission of tularemia and a wide range of animal hosts, including small mammals, birds, and humans, are susceptible to infection by *F. tularensis* [3]. Besides possessing a wide range of hosts, the pathogen is also quite resilient to environmental conditions and has been found to survive in mud or water for over a year [4].

Several studies have shown that *F. tularensis* pathogenicity is dependent on its ability to persist and replicate inside host cells, especially macrophages [1], [5]. Several of the genes required for virulence are located within the *Francisella* pathogenicity island (FPI), though additional genes are likely to be required [6]. A number of genes in the FPI, such as *pdpA* and *pdpB*, have been shown to be required for intracellular bacterial growth and survival [7], [8]. Genes outside the FPI have also been implicated in aspects of *F. tularensis* virulence and dissemination through the utilization of genome-wide screens [8], [9]. While a limited number of *F. tularensis* regulators are known, those shown to be involved in virulence include MglA, SspA, FevR, and RelA [10], [11], [12]. Recently, the *F. tularensis* subspecies *holarctica* strain LVS Hfq protein was reported to play a role in virulence, possibly by negatively regulating the *pdp* operon of the FPI [13].

The RNA-binding protein Hfq is an important regulator of gene expression in a number of bacterial pathogens including *Yersinia pseudotuberculosis*, *Neisseria meningitidis*, and *Salmonella enterica* [14], [15], [16]. The Hfq protein was first identified as a host factor required for synthesis of bacteriophage Qβ RNA in *Escherichia coli* and belongs to the Sm and Sm-like family of proteins found in both prokaryotes and eukaryotes [17], [18]. Hfq is present in many bacterial species and *hfq* mutants often exhibit pleiotropic phenotypes including defects in virulence, quorum sensing, growth rate, and stress tolerance [19], [20], [21], [22], [23].

Recent studies suggest that Hfq acts primarily as a post-transcriptional regulator that facilitates RNA-RNA interactions between small noncoding RNAs (sRNAs) and their mRNA targets [18], [24]. Small RNAs are a novel group of gene regulators that typically act by base pairing with target mRNAs resulting in either the up- or down-regulation of protein synthesis. These sRNAs are often encoded in *trans* and commonly rely on the Hfq protein for mediating the sRNA-mRNA interaction [25].

Because the genomes of *Francisella* species encode only one alternative sigma factor, σ^32^, and one complete two-component regulatory system [26], [27], the Hfq protein of the mouse pathogen *F. novicida* was targeted in an effort to discover new regulatory mechanisms employed by *Francisella* for stress survival. *F. novicida* is closely related to *F. tularensis* but is unable to cause disease in healthy individuals, though it does cause a disease in mice that is very similar to human tularemia [7]. As such, it provides an alternative model system for tularemia work. Here we report the characterization of two separate *F. novicida hfq* transposon insertion mutants, a complete *hfq* deletion mutant, and the heterologous expression of the *F. novicida* Hfq in an *E. coli hfq* mutant. We demonstrate that the N-terminal half of the protein is most critical to heat stress tolerance and that the entire protein is unable to complement the phenotype of an *E. coli hfq* mutant. Not only does Hfq play a role in the response to salt and heat shock, as reported for *F. tularensis* subspecies *holarctica* [13], it also plays a role in pH tolerance, peroxide stressors, entry into stationary phase, and biofilm formation in *F. novicida*. We also report the expression profile of a subset of genes previously identified by Meibom and coworkers to be regulated by Hfq in *F. tularensis* subspecies *holarctica* [13]. Thus Hfq and likely sRNAs appear to play a role in regulating global gene expression, stress resistance, and formation of biofilms in *F. novicida*.

## Materials and Methods

### Bacterial Strains and Growth Conditions

Bacterial strains and plasmids used in this study are listed in Table 1. *F. novicida* strain U112 and all its derivatives were routinely grown at 37°C in Tryptic Soy (TS) broth (Difco) supplemented with 0.1% L-cysteine or on TS agar plates supplemented with 0.1% cysteine, unless otherwise noted. *E. coli* strains were grown in Luria-Bertani (LB) medium or on LB agar plates. When required, media was supplemented with chloramphenicol (50 µg/ml), erythromycin (250 µg/ml), kanamycin (50 µg/ml), or tetracycline (10 µg/ml).

**Table 1:** Bacterial strains and plasmids used in this study.

Strains	Genotype or description	Source or reference
*F. novicida*		
U112	Wild-type *F. tularensis* subsp. *novicida* strain U112	[56]
U112 Δ*hfq*	U112 Δ*hfq*, Em^R^	This study
U112 Tn*hfq*1	U112 *hfq* :: EZ-Tn5 <KAN-2> at ORF position 229 of 327 bp	[37]
U112 Tn*hfq*2	U112 *hfq* :: EZ-Tn5 <KAN-2> at ORF position 133 of 327 bp	[37]
*E. coli*		
MC4100	*araD139* Δ(*argF-lac*)*205 flb-5301 pstF25 rpsL150 deoC1 relA1*	[24]
GS081	MC4100 *hfq*-1 :: Ω(Cm^R^)	[24]
**Plasmids**		
pKK214*gfp*	Low-copy-number expression vector with *groEL* promoter of *F. tularensis* LVS, Δ*cat::gfp*	[30]
pKK214*hfq*	*hfq* ORF and 191 bp-upstream region inserted into pKK214*gfp*	This study
pACYC177	Low-copy-number cloning vector, Amp^R^, Km^R^	New England Biolabs
pACYCMc*hfq*	MC4100 *hfq* ORF and 163 bp upstream region inserted into pACYC177	This study
pACYCFn*hfq*	U112 *hfq* ORF and MC4100 *hfq* promoter region region inserted into pACYC177	This study
pIDN4	Source of erythromycin gene cassette	[29]
pMAL-c4X	Vector for protein purification containing maltose binding protein	New England Biolabs

### Strain construction

To construct a non-polar *F. novicida hfq* deletion mutant, a 3,033 bp DNA cassette containing an *ermC* ORF flanked by 1,170 bp upstream and 1,128 bp downstream of the *hfq* (FTN_1051) ORF was constructed via fusion of three separate PCR products [28]. Primers for the cassette contained overlapping sequences and are listed in Table 2. The following PCR reaction mixture was used to amplify the upstream (primers FnHfqKO-upF/FnHfqKO-upR) and downstream (primers FnHfqKO-downF/FnHfqKO-downR) regions flanking the *hfq* ORF from *F. novicida* as well as the 735 bp *ermC* ORF from pIDN4 [29] (primers FnHfqKO-ermF/FnHfqKO-ermR): 1× *PfuUltra™* II Buffer (Stratagene), 0.25 mM each dNTP, 2.5 pmol each primer, 1 µl *PfuUltra™* II Fusion HS DNA polymerase (Stratagene), and 1 µl template DNA in a total volume of 50 µl. Reactions were cycled according to the following program: 95°C denaturation for 2 min; followed by 6 cycles consisting of 95°C for 30 sec, 45°C for 30 sec, and 72°C for 70 sec; then 25 cycles consisting of 95°C for 30 sec, 54°C for 30 sec, and 72°C for 70 sec; and ending with a 6 min extension at 72°C. The resulting PCR products were gel purified using the QiaQuick Gel Extraction kit (Qiagen).

**Table 2:** Primers used in this study.

Primer name	Primer sequence (5′ - 3′)
FN_hfqProm-F-SmaI	TTTCCCGGGATGGAGGACGAAACTAAAGAGTTAGATAT
FN-hfq.orf-EcoRI-R	CGGAATTCTTACTCGTGAATATTACCTTCATTC
FN_hfq-R-PstI	GCAATCTGCAGTTACTCGTGAATATTA
FN_hfq-22F	CAAGACCCGTTCTTAAATGC
FN_hfq-326R	TCGTGAATATTACCTTCATTCTC
FN_hfq-151R	AGCTGGAACTATAGTAGAAATAGCATGT
FN-hfq-ATG-F	ATGTCAAGAATATCATCTTTACAAGACC
FN-hfq-330R-PstI	GCAATCTGCAGTTACTCGTGAATATTA
FnHfqKO-ermF	TAGATTCGAGGTACGGCTACAGTCTTTTGGCTAACACACACGCCATTC
FnHfqKO-ermR	CGGTCTATGAACTTAGTGAGCGGATTAGTTTATGCATCCCTTAACTTACTTA
FnHfqKO-upF	CTATGGTTTGGCTGGACCAACAGCTTC
FnHfqKO-upR	AAGACTGTAGCCGTACCTCGAATCTATGTCTCACTTCCTTTTAA
FnHfqKO-downF	AATCCGCTCACTAAGTTCATAGACCGAGTTCCATTGTGGAGTAATATTAG
FnHfqKO-downR	TCCTCAAGAGGCACGAAACTTGGC
FN_hflX-373F	AAACTTCAGGTTGAGTTGGCGCAG
FN_hflX-1013R	TCAAGAGGCACGAAACTTGGCTTG
FN_hflX-501R	TATCTCAAGCTGTGTCTCACCAGGTC
FN_miaA-25F	GCTGGACCAACAGCTTCAGGTAAA
FN_miaA-274F	GGAAGAGAAGTCTTACTTGTTGGTGGG
FN_miaA-833F	CATGGATTCGTAATTGGCAGAG
Q_FN-bioD-111F	ACCTGTAGCGTCTGGACAAAGTCA
Q_FN-bioD-241R	GAGGAGCAACTGCTTGATTGAATG
Q_FN-dnaG-859F	GCTGCAGTAGCTACATTAGGCACA
Q_FN-dnaG-966R	TTGCCCTGCTTCATCACCATCA
Q_Fn_hfq-84F	CGGGATCAAACTACAAGGTCAAGT
Q_Fn_hfq-195R	AGCTGGAACTATAGTAGAAATAGCATGT
Q_FN-katG-798F	AACTCATGGTGCAGTTCCAGAGGA
Q_FN-katG-895R	TATTGTGCCAGCCTAGACCTTGCT
Q_FN_mglA-156F	ACCTACGCTTAGCACAGATGA
Q_FN_mglA-249R	TGGAAACATCGGAGGAAAGGGA
Q_FN-pdpA-692F	ACGGCATAAACGGCTGGTTAACTT
Q_FN-pdpA-787R	CAAGCAATATGGGTTGATTTGGGC
Q_FN-pdpB-2681F	ACTCGGCTGCAACAAATGAAGC
Q_FN-pdpB-2767R	GTGGAGATAGCTGCTCTATAAATCCAGAGT
Q_FN-pyrF-485F	ATGTCCCAGGTGTAAGGCTTGAGA
Q_FN-pyrF-604R	CGCGTAATAGCGGCCTACCTACAATA
Q_FN-relA-1042F	ACAGTTGTCAAAGTTGGCGAGCAG
Q_FN-relA-1148R	CCTTCTTTATAACGCCAATGTGCCGC
Q_FN-uvrD-140F	GTCGTGATAAAGGCGTATCTGTGG
Q_FN-uvrD-226R	TTTCTACACGCTGCTGGATCTC
Q_FN-yhbG-183F	AGTACGCATGGGTCAAGAGGATGT
Q_FN-yhbG-273R	AACCGAAGCCTCCTGAGGCAAATA
Ec_hfq-R-PstI	GCAATCTGCAGTTATTCGGTTTCTTCGCTG
Ec_hfq-up-F-BamHI	CTTGGATCCTTCACTGGCTTGACAGTGAAAAACCAGAAC
EcFnHfq-FusF	GCATATAAGGAAAAGAGAGAATGTCAAGAATATCATCTTT
EcHfqProm-FusR	AAAGATGATATTCTTGACATTCTCTCTTTTCCTTATATGC

For the initial fusion reaction, 100 ng of each purified product was added to a PCR mixture as described above except no primers were added. The products themselves were used as primers. This initial fusion mixture was cycled according to the following program: 95°C denaturation for 15 sec; followed by 5 cycles consisting of 95°C for 15 sec, 55°C for 60 sec, and 72°C for 210 sec. The final fusion reaction contained 20 µl of the initial fusion product, 2.5 pmol each of primers FnHfqKO-upF and FnHfqKO-downR, 1× *PfuUltra™* II Polymerase Buffer, 0.25 mM each dNTP, and 1 µl *PfuUltra™* II Fusion HS DNA polymerase. This reaction was cycled using the following parameters: 25 cycles consisting of 95°C for 15 sec, 55°C for 60 sec, and 72°C for 210 sec; and ending with a 8 min extension at 72°C. Sequence analysis of the final fusion product was used to verify lack of sequence errors and that the *hfq* ORF was replaced with the *ermC* ORF.

To construct the complementing plasmid pKK214*hfq*, the *hfq* coding region and 191 bp directly upstream were PCR amplified from *F. novicida* using primers FN_hfqProm-F-SmaI and FN-hfq.orf-EcoRI-R using a standard reaction mixture and cycling parameters. The product was digested with SmaI and EcoRI and ligated into SmaI-EcoRI-digested pKK214*gfp* [30], producing pKK214*hfq*. All constructs were verified by DNA sequence analysis and introduced into the appropriate *F. novicida* strains by electroporation [31]. Transformants were screened and confirmed by PCR analysis and DNA sequencing, and the resulting mutant strains were designated *F. novicida* Δ*hfq* and complemented strain Δ*hfq*/pKK214*hfq*.

To complement an *E. coli hfq* mutant with the *F. novicida* Hfq protein under control of the *E. coli hfq* promoter, the wild-type *F. novicida hfq* ORF was PCR amplified using primers EcFnHfq-FusF and FN_hfq-R-PstI (Table 2) in a standard reaction mixture. The 163 bp promoter region of the native *E. coli hfq* ORF was amplified with primers Ec_hfq-up-F-BamHI and EcHfqProm-FusR. The two PCR products were ligated together using a PCR fusion reaction as described above. The PCR product was digested with PstI and BamHI and ligated into pACYC177 that had been digested with the same restriction enzymes, and then transformed into the *E. coli hfq* mutant strain GS081 [24]. Kanamycin-resistant transformants were screened for the presence of *F. novicida hfq* linked to the *E. coli hfq* promoter region by PCR and analyzed by DNA sequence analysis. One transformant was selected for further characterization; its plasmid designated pACYC177Fn*hfq*. The presence of *F. novicida* Hfq protein was verified by Western blot analysis using the method described below. Construction of the control plasmid pACYC177Mc*hfq* containing the native *E. coli hfq* ORF and 163 bp upstream was performed as described above using primers Ec_hfq-R-PstI and Ec_hfq-up-F-BamHI. Complementation was determined by phenotypic analysis at 37°C in LB plus appropriate antibiotics as described above.

### Phenotypic analyses

Strains were grown overnight at 37°C in TS broth supplemented with 0.1% cysteine and the appropriate antibiotic. The cultures were diluted 1000-fold in fresh TS broth with cysteine and appropriate antibiotics, supplemented with the appropriate stressor and grown at 37°C (42°C for heat shock) with shaking at 200 rpm. Optical density was measured at 600 nm. For stress tolerance tests the following conditions were used: 1% and 2% additional NaCl, medium adjusted to pH 5, 0.0015% hydrogen peroxide, and heat shock at 42°C. All phenotypic analyses were performed at least two times and the results of a single representative experiment are presented.

### Antibody production and immunoblot analysis

The coding sequence for Hfq was PCR amplified from *F. novicida* using PfuUltra II Fusion HotStart Polymerase (Agilent Technologies) and primers FN-hfq-ATG-F and FN-hfq-330R-PstI (Table 2) at 50°C annealing. The resulting blunt-ended PCR product was digested with PstI, gel purified, and cloned into the XmnI and PstI sites of the expression vector pMAL-c4X (New England BioLabs), which contains a cleavable N-terminal maltose-binding protein ORF. The tagged Hfq was expressed in DE3 cells via IPTG induction, purified using amylose resin, and cleaved from the maltose-binding protein by Factor Xa as described in the pMAL Protein Fusion and Purification System manual (New England BioLabs). An equal volume of SDS sample buffer (40 mM Tris-HCl, pH 6.8, 2% sodium dodecyl sulfate [SDS], 1% β-mercaptoethanol, 10% glycerol, 0.4 mg/ml bromophenol blue) was added to the resulting digest, boiled at 95°C for five minutes, and separated by SDS-PAGE (12% gel). A ∼13 kD band corresponding to the Hfq protein was excised and sent for commercial anti-sera production (Cocalico Biologicals).

For immunoblot analysis, *F. novicida* and *E. coli* strains were grown to exponential phase in TS broth supplemented with 0.1% cysteine and LB, respectively, with appropriate antibiotics at 37°C; equivalent units of optical density at 600 nm (OD_600 nm_) were taken from each culture, resuspended in SDS sample buffer, boiled at 95°C for five minutes, and separated by SDS-PAGE (12% gel). After electrophoresis, proteins were transferred to a PVDF membrane (Millipore) as described by the manufacturer using a Trans-Blot apparatus (Bio-Rad) at 350 mA for 1 h. The resulting membrane was blocked for 1 h and then incubated overnight with *F. novicida* Hfq antiserum. After washing and blocking, a horseradish peroxidase-linked secondary antibody (Thermo Scientific) was added and additional washing steps were performed. Final binding was detected using SuperSignal West Pico Chemiluminescent substrate (Thermo Scientific).

### Microtiter plate biofilm production assay

Crystal violet assaying for biofilm formation was performed as previously described [32]. *Francisella* strains were grown overnight at 37°C in either Mueller-Hinton (MH) broth or TS broth supplemented with cysteine. Cultures were diluted 1∶100 in fresh medium and vortexed. After vortexing, 150 µl volumes were aliquoted per well in a 96-well polystyrene plate, previously rinsed with 70% ethanol and air-dried. The bacteria were grown statically at 37°C for 48 hours. Wells were washed three times with distilled water, allowed to dry, and then 200 µl of 0.1% crystal violet was added to each well for 45 min of incubation. Wells were washed five times with distilled water, allowed to dry, and the remaining biomass that absorbed crystal violet was solubilized with 95% ethanol. Staining was then quantified by obtaining the OD_590 nm_ using a Nano-Drop spectrophotometer (Thermo Scientific) [33].

### RNA isolation and techniques

Cultures of U112 and its Δ*hfq* derivative were grown to exponential (OD_600 nm_ 0.3–0.6) and stationary phase (OD_600 nm_>0.8) in TS broth supplemented with cysteine. Prior to nucleic acid extraction, a 1/5 volume of ethanol/5% phenol at −20°C was added as a stop solution to prevent transcriptional changes during cell harvesting. Total RNA was isolated using TRI Reagent (Ambion) followed by treatment with TURBO DNase (Ambion) and precipitation with 3 M sodium acetate (pH 5.2) and 100% ethanol. To perform reverse transcription (RT)-PCR, 10 pmol of corresponding reverse primer (Table 2) was allowed to anneal to 1 µg of RNA and heated for 5 min at 70°C. To initiate cDNA synthesis, the following was added to the above mixture to yield a 20 µl (total volume) reaction: 1 mM mixture of dNTPs, 40 U of Ribolock RNase Inhibitor (Fermentas), 3 mM MgCl_2_, 1 µl ImProm-II reverse transcriptase (Promega), and 5× reaction buffer. The reaction was then heated for 5 min at 25°C, 60 min at 42°C, and 15 min at 70°C. Then 5 µl of the cDNA reaction mixture was used as a template in a 50 µl PCR amplification reaction mixture with corresponding forward and reverse primers (Table 2) and GoTaq DNA polymerase (Promega), as described by the supplier. For control reactions, RNA without reverse transcriptase or chromosomal DNA was used as a template.

5′ RACE was performed on 5 µg DNase-treated RNA using the GeneRacer Kit (Invitrogen) according to the manufacturer's instructions except for dephosphorylation with calf intestinal phosphatase, which was omitted. RT-PCR was performed using the FN_hfq-326R primer (Table 2) and AMV reverse transcriptase as described above. The cDNA was then amplified by PCR using GoTaq DNA Polymerase (Promega) along with FN_hfq-326R (Table 2) and the provided 5′ GeneRacer primer. PCR products were cloned into pGEM-T (Promega) and the resulting plasmids were transformed into Top10 cells. At least five transformants harboring cDNA-containing plasmid were analyzed by DNA sequencing.

### Quantitative RT-PCR (qRT-PCR)

*F. novicida* and its Δ*hfq* derivative were grown to exponential phase in TS broth supplemented with cysteine at 37°C. A portion of culture was removed for RNA extraction as described above and used as time point zero. The remaining culture was divided in half. One portion was grown in TS broth supplemented with cysteine at 37°C, while the other portion was grown at particular stress conditions as described above. Portions of both cultures were removed at 20 minutes, one hour, and four hours and RNA was extracted as described above. One microgram of DNase-treated RNA was reverse transcribed using SuperScript III First-strand synthesis SuperMix (Invitrogen) according to the protocol provided by the manufacturer. Quantitative RT-PCR was performed with SYBR green dye (Quanta Biosciences) in a MiniOpticon thermocycler (Bio-Rad) using gene-specific primers (Table 2). To calculate reaction efficiency of each gene-specific primer set, a standard curve using a series of diluted cDNA was generated. Expression of *hfq* was determined by comparing samples grown in the presence and absence of a specified stress condition. To compare transcript levels, the amounts of transcript were normalized using the DNA helicase gene *uvrD* (FTN_1594) (Fig. S1) and fold changes were calculated using the Pfaffl method in CFX Manager software (Bio-Rad) [34].

## Results

### Transcriptional analysis of *hfq* ORF

**Figure 1:**
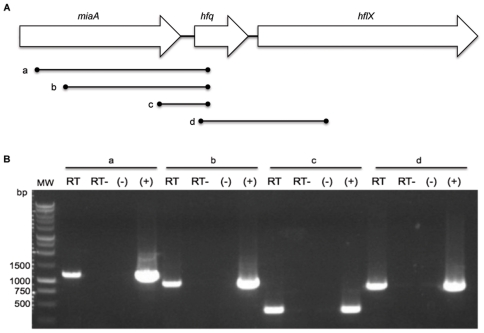
Transcription of the *hfq* locus. (A) Genetic organization of the *hfq* locus. Lines a, b, c, and d indicate regions of the locus amplified by PCR as shown in panel B. (B) RT-PCR of the *hfq* locus. Lanes 1–4 corresponds to line a, lanes 5–8 to line b, lanes 9–12 to line c, and lanes 13–16 to line d. Within each set of 4 wells, PCR to amplify lines a–d was performed with RNA after RT, RNA without RT as a control, PCR without DNA template as a control, and PCR with genomic DNA from U112 as a positive control. MW denotes molecular weight marker (1 kb BenchTop marker, Promega).

While the *F. tularensis* LVS *hfq* gene locus, promoter region, and corresponding protein sequence have previously been described [13], it was unknown if the *F. novicida* homolog possessed the same characteristics. The *F. novicida hfq* gene (FTN_1051) is located on the negative strand at bps 1110271–1110600 of the genome. The gene encodes a 109 amino acid protein and is 99.39% identical to its *F. tularensis* LVS homolog at the nucleotide level and 100% at the amino acid level. In both species, the *hfq* gene is flanked by the *miaA* gene upstream [encoding tRNA delta(2)-isopentenylpyrophosphate transferase] and *hflX* downstream [encoding a GTP-binding protein] (Fig. 1A) [13], a configuration found in a number of bacteria such as *E. coli* that may form part of an operon [35].

To test if *F. novicida hfq* was part of an operon we performed RT-PCR experiments targeting the intergenic regions directly up- and downstream of *hfq*. Transcription of *hfq* in *E. coli* has been shown to initiate from multiple upstream promoter sequences, including two located within the *miaA* gene [36]. To test if *hfq* transcription in *F. novicida* originated from other promoters besides the single one identified 65 bp upstream of the *F. holarctica hfq* gene [13], RT-PCR was performed using reverse primer FN-hfq-151R and forward primers FN_miaA-25F, FN_miaA-274F, and FN_miaA-833F (designated lines a, b, and c respectively) internal to *miaA* (Fig. 1A). The resulting PCR products (1164 bp, 915 bp, and 357 bp) indicated co-transcription of *hfq* and *miaA* throughout the length of the *miaA* ORF, suggesting at least one additional promoter region is responsible for *hfq* transcription (Fig. 1B). 5′ RACE experiments placed the start site of *hfq* transcription −65 bases relative to its ATG start codon, which matches similar work performed in *F. tularensis* LVS [13]. However, 5′ RACE did not indicate additional transcription start sites further upstream. In order to ascertain if *hfq* is co-transcribed with the downstream gene *hflX*, RT-PCR using primers FN_hfq-22F and FN_hflX-501R (line d) was performed (Fig. 1A). An 853 bp product resulted, suggesting co-transcription of *hfq* with the downstream gene *hflX* (Fig. 1B).

### *F. novicida* Hfq is unable to complement an *E. coli hfq* mutation

**Figure 2:**
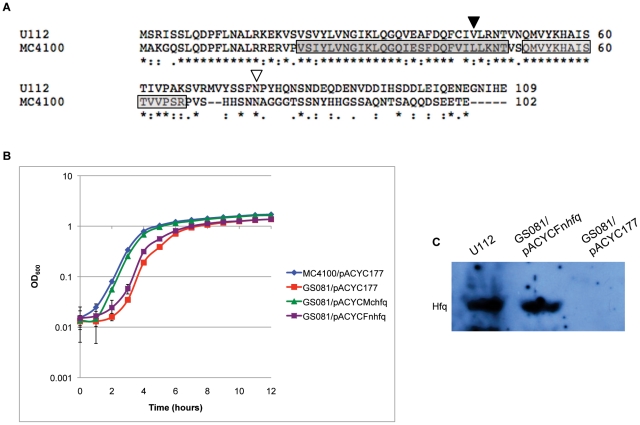
Complementation of *E. coli hfq* mutant with *F. novicida* Hfq. (A) Alignment of the Hfq proteins from *F. novicida* strain U112 and *E. coli* strain MC4100 using the ClustalW program. Periods designate semiconserved substitutions, colons designate conserved substitutions, and asterisks designate residues found in both strains. A dark gray and light gray box indicates the location of *E. coli* Sm1 and Sm2 sequence motifs respectively. Inverted arrows designate the location of transposon insertion within *F. novicida hfq*. The arrow at position 45 a.a. (▾) corresponds to strain Tn*hfq*2 insertion, and the arrow at position 76 a.a. (▿) corresponds to strain Tn*hfq* 1 insertion. (B) Growth of *E. coli* strains MC4100/pACYC177, GS081/pACYC177, GS081/pACYCMc*hfq*, and GS081/pACYCFn*hfq* in LB broth at 37°C with appropriate antibiotics. (C) Detection of *F. novicida* Hfq assessed by immunoblot analysis in *F. novicida* strain U112, GS081/pACYCFn*hfq*, and GS081/pACYC177 using anti-Hfq antisera.

The *F. novicida* Hfq protein is slightly larger than the Hfq protein of *E. coli* strain MC4100 (109 a.a. versus 102 a.a.) and is 39% and 49% identical at the nucleotide and amino acid level respectively (Fig. 2A). To investigate the functionality of *F. novicida* Hfq in the *E. coli hfq* mutant strain GS081, a region spanning the *hfq* gene was ligated to the *E. coli hfq* promoter region, cloned into plasmid pACYC177 and transformed into GS081 [24] yielding strain GS081/pACYCFn*hfq*. A region containing the native *E. coli* strain MC4100 *hfq* gene and promoter region was also cloned into pACYC177 as a control yielding pACYCMc*hfq*, which was transformed into GS081. These two transformants, along with the *E. coli* parent strain MC4100 and *E. coli hfq* mutant strain GS081, were each grown in LB broth plus appropriate antibiotics at 37°C to allow comparison of their growth rates. Additionally, MC4100 and GS081 were each grown harboring empty pACYC177 as a control, both of which displayed the same growth rate as the parent strains (data not shown). The *E. coli hfq* mutant GS081 showed a modest growth defect relative to the parent strain. Expression of *E. coli* Hfq, under the control of its native promoter, almost fully restored normal growth. However, the strain containing *F. novicida hfq* exhibited a slower growth rate than the strain containing native MC4100 *hfq* and did not appear to complement the *hfq* mutant phenotype (Fig. 2B). Non-quantitative immunoblot analysis verified expression of *F. novicida* Hfq protein in the *E. coli* mutant strain GS081 (Fig. 2C).

### Phenotypic analysis of *hfq* transposon insertion mutants

**Figure 3:**
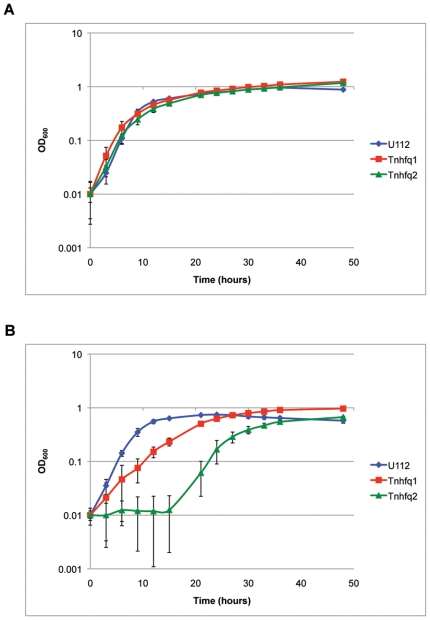
Growth characterization of *hfq* transposon strains. (A) *F. novicida* strains U112, Tn*hfq*1, and Tn*hfq*2 were cultured in TS broth supplemented with cysteine and appropriate antibiotics at 37°C and the OD_600 nm_ of each culture was measured at select time points. (B) Growth characteristics in TS broth supplemented with cysteine at 42°C.

To determine what effect interruption of the *hfq* gene has on the *F. novicida* phenotype, two separate transposon insertion mutants were obtained from a *F. novicida* library created by Gallagher and co-workers [37]. Locations of the EZ-Tn5<KAN-2> insertions mapped to positions 229 and 133 bp relative to the 327 bp *hfq* ORF for strains Tn*hfq*1 and Tn*hfq*2, respectively (Fig. 2A). Phenotypic analysis of these two *hfq* insertion mutants compared to U112 at 37°C indicated no growth difference for Tn*hfq*1 or Tn*hfq*2 (Fig. 3A). Further analysis using 42°C heat stress showed a slower rate of growth for Tn*hfq*1 compared to U112 at 42°C and its own growth phenotype at 37°C. A more severe defect in growth rate was observed for Tn*hfq*2, which took over fifteen hours to exit lag phase (Fig. 3B). The different phenotypes observed between Tn*hfq*1 and Tn*hfq*2, suggests that the N-terminal half of the *F. novicida* Hfq is more important for growth during growth in certain stress conditions than the C-terminal half.

### Construction of *F. novicida hfq* deletion mutant and growth *in vitro*

**Figure 4:**
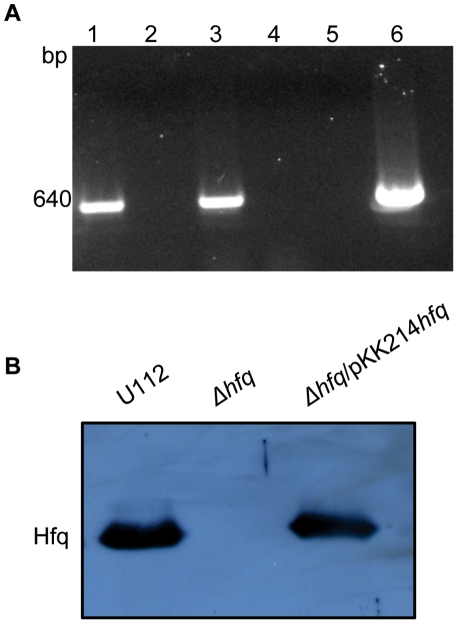
Affect of *hfq* deletion on *hflX* expression and Hfq protein expression in *F. novicida* strains. (A) RT-PCR using primers internal to *hflX* was performed with RNA isolated from U112 with RT (lane 1), RNA from U112 without RT (lane 2), RNA isolated from the *hfq* mutant with RT (lane 3), RNA from the *hfq* mutant without RT (lane 4), PCR with no DNA template as a negative control (lane 5), and PCR with DNA isolated from U112 as a positive control (lane 6). (B) Immunoblot analysis of U112, the *hfq* mutant, and *Δhfq*/pKK214*hfq* strains using anti-Hfq antiserum.

Homologous recombination was used to create a deletion mutant in which the *F. novicida hfq* ORF was replaced by an *ermC* ORF. After verifying that marker exchange had occurred by PCR and Southern blotting (data not shown), RT-PCR using primers FN_hflX-373F and FN_hflX-1013R internal to *hflX* verified that deletion of *hfq* did not have a polar effect on transcription of the downstream gene *hflX* (Fig. 4A). We then used the low copy-number vector pKK214*gfp* to generate a plasmid-based complementing clone of *hfq* under the control of its native promoter, creating strain Δ*hfq*/pKK214*hfq*. Subsequent non-quantitative immunoblot analysis verified the production of Hfq protein in both the wild-type U112 and the complemented strain, and the loss of Hfq in the mutant (Fig. 4B). Immunoblot analysis of the *hfq* mutant strain harboring empty pKK214 as a control also exhibited a loss of Hfq (data not shown).

**Figure 5:**
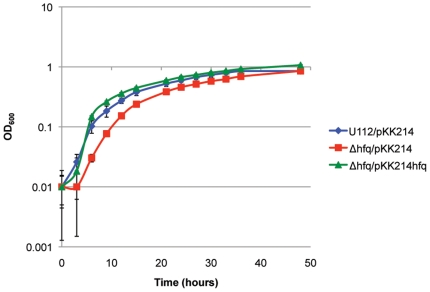
Growth characteristics of the U112 *hfq* mutant. *F. novicida* strains U112/pKK214, *Δhfq*/pKK214, and *Δhfq*/pKK214*hfq* were cultured in TS broth supplemented with cysteine and appropriate antibiotics at 37°C and the OD_600 nm_ of each culture was measured over the course of time.

To examine if the entire Hfq protein is important for *F. novicida* growth, we compared the growth of U112, Δ*hfq*, and Δ*hfq*/pKK214*hfq* strains in liquid medium at 37°C. Both the wild-type and *hfq* mutant were grown with empty pKK214 vector to account for any plasmid maintenance effects in the complemented strain. The deletion strain, Δ*hfq*/pKK214, exhibited a longer lag phase compared to wild-type U112/pKK214. The complemented strain, Δ*hfq*/pKK214*hfq*, exhibited a similar growth compared to U112/pKK214 but eventually reached a higher final optical density (Fig. 5).

### Hfq is important for certain *F. novicida* stress responses

**Figure 6:**
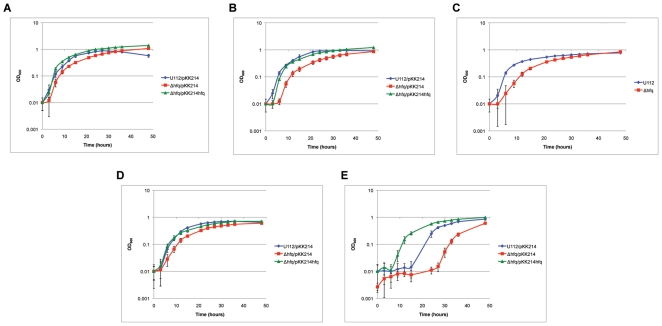
Stress resistance of the U112 *hfq* mutant. Growth characteristics of *F. novicida* strain U112/pKK214, *Δhfq*/pKK214, and *Δhfq*/pKK214*hfq* strains in TS broth supplemented with cysteine and appropriate antibiotics under the following conditions: (A) additional 1% NaCl at 37°C, (B) additional 2% NaCl at 37°C, (C) adjusted to pH 5 at 37°C, (D) at 42°C, and (E) addition of 0.0015% H_2_O_2_ at 37°C.

*Francisella* possesses a diverse lifestyle and like most pathogens is exposed to various stressors. We therefore examined if Hfq is important in the stress resistance of *F. novicida* by subjecting U112/pKK214, Δ*hfq*/pKK214, and Δ*hfq*/pKK214*hfq* strains to certain stress conditions during growth in liquid medium. When adding 1% NaCl to the medium, the U112/pKK214 strain exhibited an enhanced growth rate and reached a higher final optical density than in medium lacking this supplement, however a rapid decrease in cell density quickly followed. Growth of the Δ*hfq*/pKK214 strain was also slightly enhanced with additional 1% NaCl compared to growth in medium without this supplement (Figs. 5 and 6A). In medium containing 2% NaCl, Δ*hfq*/pKK214 exhibited a prolonged lag phase relative to medium containing 1% NaCl (Fig. 6B). Upon entry into stationary phase, U112/pKK214 cell density decreased while the *hfq* mutant remained unchanged compared to growth in unadjusted medium (data not shown). The growth of the strains was also compared in medium adjusted to pH 5. Strain U112 exhibited a slightly slower rate of growth and reached a lower final optical density compared to unadjusted medium. The Δ*hfq* strain also showed a slower growth rate and prolonged lag phase in the acidic medium compared to unadjusted medium but still reached a higher density than U112 (Fig. 6C). Growth of Δ*hfq*/pKK214*hfq* and pKK214-containing control strains exhibited little or no growth during the initial 24 hours of incubation at acidic pH (data not shown). At 42°C, the U112/pKK214 rate of growth was unchanged but the final density was lower compared to growth at 37°C. The Δ*hfq*/pKK214 strain exhibited a slightly slower rate of growth at 42°C. The Δ*hfq*/pKK214 strain also reached a lower final optical density and never surpassed the density of U112/pKK214 (Fig. 6D). Addition of 0.0015% hydrogen peroxide led to a prolonged lag phase for both U112/pKK214 and Δ*hfq*/pKK214, after which both strains exhibited a rate of growth similar to incubation in unadjusted medium. However, the complemented strain Δ*hfq*/pKK214*hfq* was able to exit lag phase sooner than even U112/pKK214 (Fig. 6E).

**Figure 7:**
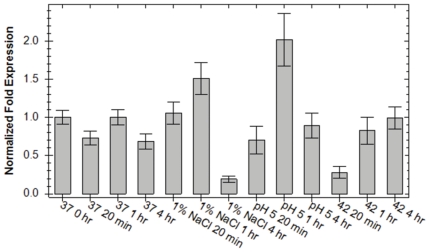
Quantification of *hfq* expression during stress exposure using quantitative real-time PCR. Normalized fold expression of *hfq* transcripts in U112 grown in TS broth supplemented with cysteine at 37°C, at 37°C with the addition of 1% NaCl, at 37°C in medium adjusted to pH 5, and in unadjusted medium at 42°C. Transcript levels were normalized to the level of DNA helicase gene expression (FTN_1594).

To further examine the role of Hfq in the stress response of *F. novicida* we monitored *hfq* expression using qRT-PCR experiments. Cultures of U112 were grown to exponential phase, then separated into two portions as described in the methods. One portion was allowed to continue growth at 37°C while the other was incubated with additional 1% NaCl, in medium adjusted to pH 5, or in unadjusted medium at 42°C. Expression of *hfq* was quantified (using primers Q_Fn_hfq-84F/Q_Fn_hfq-195R) from RNA extracted at 20 minutes, one hour, and four hours post-separation and compared to *hfq* levels from time 0 (pre-separation). Measurements of culture optical density at these time points indicate an approximate correlation to exponential, early stationary, and late stationary phases respectively. Expression of *hfq* exhibited a 5.21-fold decrease after prolonged growth (4 hr) in medium containing additional 1% NaCl. A similar decrease (3.55-fold) in *hfq* expression was observed shortly after incubation at 42°C. Conversely, a 2.02-fold increase in expression was observed after one hour of growth in acidic medium. We did not observe significant changes in *hfq* expression for the other measured time points (Fig. 7).

### Hfq promotes formation of biofilms in *F. novicida*

**Figure 8:**
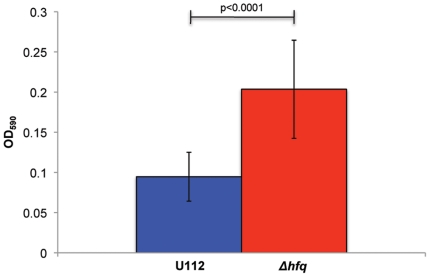
Biofilm formation by *F. novicida* U112 and *Δhfq* strains. Both strains were assayed for biofilm formation using crystal violet staining after 48 hours of static growth in MH broth at 37°C. Culture OD_590 nm_ was determined for multiple replicates. U112 demonstrated decreased crystal violet straining compared to *Δhfq* (p<0.0001).

Biofilm growth has been shown to confer both enhanced survival during stress conditions and environmental persistence. Because *Francisella* has been shown to produce biofilms [10], [33], [38], we decided to test biofilm formation by U112 and the Δ*hfq* strain. Cultures were grown statically in microtiter plates at 37°C for 48 hours followed by staining with 0.1% crystal violet and measurement of the OD_590 nm_. Both *F. novicida* strain U112 and the *hfq* mutant showed similar crystal violet staining when grown in TS broth supplemented with cysteine (data not shown). However, the *hfq* mutant exhibited increased crystal violet staining compared to wild-type when grown in MH broth indicating increased accumulation of adherent biomass (Fig. 8).

### Loss of Hfq results in expression changes for a variety of genes

From microarray analysis of a *F. tularensis* LVS *hfq* mutant, numerous genes were identified as being regulated by Hfq [13]. We selected genes from that study which exhibited at least a 2-fold change in expression to profile in *F. novicida* strains U112 and Δ*hfq*. Quantitative real-time PCR was performed on RNA extracted from cultures of U112 and Δ*hfq* grown to exponential and stationary phase. During exponential phase, the expression of two genes decreased significantly (*bioD* [FTN_0812] and *pdpB* [FTN_1310]). One of these genes, *pdpB*, is part of the *Francisella* pathogenicity island [7]. Four genes displayed significantly altered expression between U112 and Δ*hfq* during growth in stationary phase. Two of these genes were upregulated in the *hfq* mutant (*dnaG* [FTN_0914] and *pyrF* [FTN_0035]), while the other two genes were downregulated (*katG* [FTN_0633] and *pdpA* [FTN_1309]). Additionally, we examined expression of the *mglA* gene (FTN_1290) which encodes a global transcriptional regulator previously shown to regulate *hfq* [39]. While no significant change was found during exponential phase, expression of *mglA* was upregulated during stationary phase in the *hfq* mutant.

## Discussion

The ability to monitor environmental changes within the host organism and adjust the expression of stress- and virulence-associated genes accordingly is critical for the growth and survival of pathogenic bacteria. The importance of the small RNA chaperone Hfq in modulating such genes has been demonstrated in a variety of both intra- and extracellular bacterial pathogens [19], [20], [40]. This study demonstrates the role Hfq plays in stress tolerance, gene expression, and biofilm formation in the intracellular pathogen *Francisella novicida*.

Previously, it was shown that *hfq* transcription in *F. tularensis* LVS is controlled by a single promoter located in between the *miaA* and *hfq* ORFs [13]. Interestingly, we found that while *hfq* is transcribed from an identical promoter region in *F. novicida*, it is also co-transcribed with the upstream gene *miaA*. Thus expression of the locus in *F. novicida* appears to differ from that of *F. tularensis* LVS. Alternatively, this conflicting data could be explained by differences in experimental design. In *E. coli*, multiple upstream promoters, including two within *miaA*, drive transcription of *hfq*, which is located within the *miaA-hfq-hflX* operon [36]. This genomic locus is conserved in *F. novicida*, making it tempting to speculate that *hfq* expression is also under the control of multiple promoters both within and upstream of *miaA*. However, because our results only indicate a minimum of co-transcription with the majority of the *miaA* transcript (Fig. 1), the exact location and number of additional promoter regions remain to be determined. Despite the shared operon structure, Hfq in *F. novicida* is unable to complement an *E. coli hfq* mutant (Fig. 2B). This inability to complement an *E. coli hfq* mutant is likely due to basic structural differences between the two Hfq proteins as reflected in the amino acid sequence, especially in the two Sm-domains (Fig. 2A).

Complete deletion of *F. novicida hfq* only resulted in a modest growth defect compared to wild-type U112 (Fig. 5). The transposon mutant analysis illustrates the importance of the *F. novicida* Hfq N-terminal domain during 42°C growth (Fig. 3B). The N-terminal insertion of strain Tn*hfq*2 would disrupt a region similar to the Sm1 domain of *E. coli* Hfq, while the C-terminal disruption of strain Tn*hfq*1 would leave both Sm domains intact (Fig. 2A) [41]. The Sm motifs found in Hfq proteins are vital for hexameric formation and riboregulation, while the exact function of the C-terminal half remains unclear [22], [42]. However, stability of the two transposon mutant variants is unknown since a Western blot signal could not be observed using the *F. novicida* Hfq specific antisera (data not shown). While neither insertion strain exhibited a more dramatic phenotype than the complete *hfq* deletion strain during growth at 37°C, the lag phase of Tn*hfq*2 during heat shock was more pronounced than the *hfq* knockout strain (Fig. 3B and Fig. 6D). The reason for this discrepancy is unclear, but further studies are currently underway to elucidate this phenotype.

The importance of Hfq for stress tolerance has been reported in a variety of other bacteria, including pathogens such as *Pseudomonas aeruginosa* and *Neisseria gonorrhoeae* [43]. As such, the importance of *F. novicida* Hfq in growth and survival during stress conditions was examined by comparing phenotypic differences between the *hfq* mutant strain and wild-type U112, as well as monitoring changes in *hfq* transcription. The slightly faster growth rate for both U112/pKK214 and *Δhfq*/pKK214 when exposed to increased salt concentration suggests that additional NaCl provides a growth advantage to both strains (Figs. 5, 6A, and 6B), while the increased lag phase of *Δhfq*/pKK214 when exposed to 2% additional NaCl and the subsequent reduced transcription of *hfq* during growth in 1% NaCl (Fig. 6B and 7) indicate a potential contribution for Hfq during osmotic stress. This finding is also supported by previous work in *F. tularensis* subspecies *holarctica* [13]. Similar phenotypes have been observed in Gram-negative pathogens such as *Burkholderia cepacia*, *Moraxella catarrhalis*, and *Neisseria gonorrhoeae* [43].

Tolerance to low pH is important in *F. tularensis* due to its ability to survive and escape the phagosome during intracellular infection of certain host cell types such as macrophages. Phagosomes have previously been shown to become acidic in response to *F. tularensis* infection [44]. While both U112 and *Δhfq* exhibited a reduced rate during growth in acidic media, the prolonged lag phase of *Δhfq* (Fig. 6C) as well as increased expression of *hfq* after one hour in acidic conditions (Fig. 7) suggest a role for Hfq in the initial response to low pH. Interestingly, growth of the complemented strain and strains harboring empty pKK214 was severely inhibited by the decreased pH suggesting difficulties in plasmid maintenance during growth at pH 5, and was therefore not included in this analysis. Additionally, we examined the growth rate of U112/pKK214 and *Δhfq*/pKK214 during heat shock. Compared to the salt and low pH exposure, heat shock had a more negative effect on the growth rate of *Δhfq*/pKK214 and resulted in entry into stationary phase at a lower optical density than all other conditions tested (Fig. 6). This phenotype suggests a role for Hfq in the heat shock response even though transcription of *hfq* was initially repressed upon introduction of the stressor before returning to normal levels (Fig. 7).

**Figure 9:**
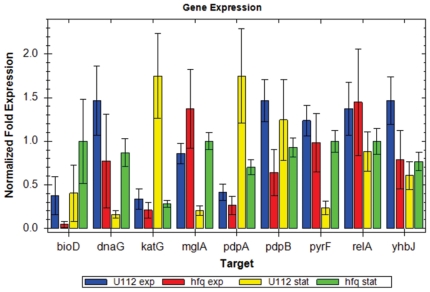
Quantification of selected gene transcription using quantitative real-time PCR. Changes in gene expression in the *hfq* mutant compared to wild-type U112 were examined during both exponential (exp) and stationary phase (stat) using RNA derived from growth in TS broth supplemented with cysteine at 37°C. Each gene was analyzed using RNA derived from three separate cultures and each reaction was performed in triplicate. Transcript levels were normalized to the level of DNA helicase (FTN_1594).

Reactive oxygen species are one defense mechanism used by macrophages, monocytes, and neutrophils to combat intracellular pathogens. *F. tularensis* has been reported to inhibit particular neutrophil killing mechanisms such as the respiratory burst as part of its infection lifecycle [45], [46]. Oxidative stress induced by addition of hydrogen peroxide had a severe effect on both U112/pKK214 and *Δhfq*/pKK214 as indicated by their pronounced lag phases (Fig. 6E). However, the additional six-hour lag phase exhibited by *Δhfq*/pKK214 indicates that Hfq is necessary for combating initial exposure to H_2_O_2_. Similar growth responses to oxidative stress have been reported for *hfq* mutants in an array of pathogens [43]. While expression of *hfq* was not assayed under this condition, expression of *mglA* was upregulated in the mutant (Fig. 9). Previous studies using a *F. novicida mglA* mutant have shown that lack of MglA results in increased resistance to H_2_O_2_ [39]. It is therefore tempting to speculate that the increased expression of *mglA* in *Δhfq* is directly linked to the increased lag phase of the strain during H_2_O_2_ exposure. However, further studies are necessary to determine the role Hfq plays in regulating *mglA* expression. As a whole, these experiments demonstrate a role for *F. novicida* Hfq during growth under a variety of environmental conditions.

The highly pleiotropic phenotype of an *E. coli hfq* mutant is largely due to the importance of Hfq in sRNA-mediated regulation. A number of sRNA targets are global regulators such as the alternative sigma factors *rpoH*, encoding the heat-shock sigma factor σ^32^, and *rpoS*, which encodes the general stress signal factor σ^S^ [47], [48]. *F. novicida* lacks an annotated *rpoS* gene and to date no known master regulator controlling the transition from exponential to stationary phase has been characterized. Interestingly, we observed the *hfq* mutant reaching a higher cell density before entry into stationary phase compared to wild-type under most conditions tested (Figs. 5 and 6). The complemented strain (*Δhfq*/pKK214*hfq*) also surpassed the cell density of U112/pKK214 before entering stationary phase. Because the native promoter region directly upstream of *hfq* is solely responsible for expression of Hfq in the complemented strain, the presence of multiple promoter regions controlling transcription of *hfq* in U112 suggests that the partial-complementation observed in *Δhfq*/pKK214*hfq* is due to insufficient cellular concentration of Hfq, especially as cellular density increases. The importance of controlling intracellular Hfq concentration has been shown in several bacteria, including *Acinetobacter baylyi*, where overexpression led to an inability of the cells to assemble into chains [49]. Additionally, while investigating the role of Hfq in *Yersinia pseudotuberculosis*, Bai and colleagues found that *hfq* mRNA levels in a partially complemented strain were lower than those of the wild-type due to differential transcription from multiple promoters. A multicopy construct was required to express *hfq* to levels necessary for full complementation to be observed [50]. Guina and coworkers have also shown that MglA is important for *F. novicida* survival during stationary-phase growth [39]. Thus the increased expression of *mglA* in *Δhfq* reported here could also be directly related to the increased cell density observed during stationary-phase. Additionally, global analysis of gene expression in a *F. tularensis* LVS *hfq* mutant strain revealed upregulation of *rpoD*, the gene encoding the exponential phase σ^70^ [13]. Farewell et al. demonstrated that overexpression of σ^70^ in *E. coli* dramatically alters gene expression and inhibits development of σ^S^-dependent phenotypes, mimicking the effect of an *rpoS* mutation [51]. Despite the lack of an annotated σ^S^ in *F. novicida*, we speculate that Hfq plays a role in the transition from exponential to stationary phase and that this role is dose-dependent.

Biofilm formation plays an important part in the transmission and environmental persistence of many bacterial pathogens. Because *F. novicida* has recently been shown to form biofilms on natural and chitin surfaces [33], we wanted to examine if Hfq contributes to biofilm formation in *F. novicida*. While no significant differences in crystal violet staining were observed between the two strains after growth in TS broth, the higher crystal violet staining exhibited by the *hfq* mutant strain when grown in the more restrictive MH broth is indicative of increased biofilm formation (Fig. 8). This level of biofilm formation was similar to that reported for *F. novicida* on chitin surfaces [33]. Biofilm formation in *Campylobacter jejuni* is significantly increased during growth in MH broth compared to more nutrient-rich media, so it is possible that *F. novicida* grown in rich TS broth is unable to form a substantial enough biofilm to observe a phenotypic difference between wild-type and the *hfq* mutant strain [52]. However, a ready explanation for increased biofilm formation in the *hfq* mutant during growth in MH broth is not apparent. Attia et al. demonstrated a growth advantage for a *Moraxella catarrhalis hfq* mutant over the wild-type in a continuous flow biofilm system. The authors suggest this phenotype results from altered gene expression in the *Δhfq* background leading to changes in the overall outer membrane architecture [53]. In accordance with this hypothesis, Meibom et al. found expression of an outer membrane protein (FTL_0535) and several Type IV pili-associated proteins (FTL_0797, FTL_0798, and FTL_0827) upregulated in a *F. tularensis* LVS *hfq* mutant strain [13]. While further expression analyses are needed to confirm this hypothesis, it seems possible that changes in outer membrane protein architecture could account for the increased biofilm formation observed in the *F. novicida hfq* mutant.

Hfq is an important regulator for a variety of genes in numerous bacteria, including *F. tularensis* LVS [13], [20], [40]. In order to evaluate its role in *F. novicida*, we monitored expression of nine genes identified by microarray analysis of a *F. tularensis* LVS *hfq* mutant [13]. Seven of these genes showed significant (greater than 2-fold) changes in expression. Two genes, *pdpA* and *pdpB*, encoded within the *Francisella* pathogenicity island were downregulated at different times in the *hfq* mutant. Previous studies have shown that inactivation of these two genes in *F. novicida* leads to growth defects within host cells and attenuation of virulence [6], [8]. Another downregulated gene, *katG*, encodes a protein annotated as possessing catalase/peroxidase activity, potentially a factor in the severe *Δhfq* growth defect observed during oxidative stress (Fig. 6E), as inactivation of *katG* increased susceptibility of *F. novicida* to hydrogen peroxide [39]. Three of the genes examined in this study, *katG*, *dnaG*, and *pyrF*, exhibited similar expression changes to those previously reported by Meibom and colleagues while three other genes, *pdpA*, *pdpB*, and *bioD*, exhibited different changes in expression [13]. While the reason behind these differences is unclear, it is interesting to note that two of the genes, *pdpA* and *pdpB*, are virulence factors encoded in the FPI, of which there is only one copy in the *F. novicida* genome compared to more virulent strains of *Francisella* which contain two copies [54]. Additionally, the transcriptional regulator MglA was upregulated during stationary phase growth in the *hfq* mutant. MglA has been implicated in coordinating both virulence gene expression and the stress response of *F. tularensis* by interacting with SspA (Stringent starvation protein A) and binding RNA polymerase [12], [39]. Absence of MglA has also been shown to result in increased *katG* expression in *F. novicida* [39], thus the downregulation of *katG* observed in this study could be a consequence of increased *mglA* expression in the *Δhfq* strain. While we are unable to determine if the expression changes observed for the profiled genes are directly or indirectly affected by Hfq, the protein still appears to play a role in their expression.

The ability of Hfq to affect such wide-ranging cellular functions is likely due to its role as an RNA chaperone for sRNAs. Hfq facilitates pairing interactions between small RNAs and their mRNA targets in several bacteria leading to post-transcriptional regulation of the target gene [55]. To date, no small RNAs have been identified in *F. novicida* so the exact regulatory function of Hfq remains to be elucidated, but it is likely that many of the effects observed here are due to interactions with as yet unidentified sRNAs. Our work demonstrates the importance of Hfq in cell growth, stress resistance, gene expression, and biofilm formation in *F. novicida*. Information from these studies is critical to understanding how this pathogen responds to and survives in the diverse environments it occupies.

## Supporting Information

Figure S1 **Expression of qRT-PCR reporter gene** ***uvrD*** **.** Relative change in *uvrD* expression across multiple time points using RNA derived from U112 growth in TS broth supplemented with cysteine at 37°C.
(TIF)
