# Supplementary figures and images for: The RNA Chaperone Hfq Is Important for Growth and Stress Tolerance in Francisella novicida
Source: PLoS One. 2011 May 5;6(5):e19797. doi: 10.1371/journal.pone.0019797 (PMC3088715; doi:10.1371/journal.pone.0019797)

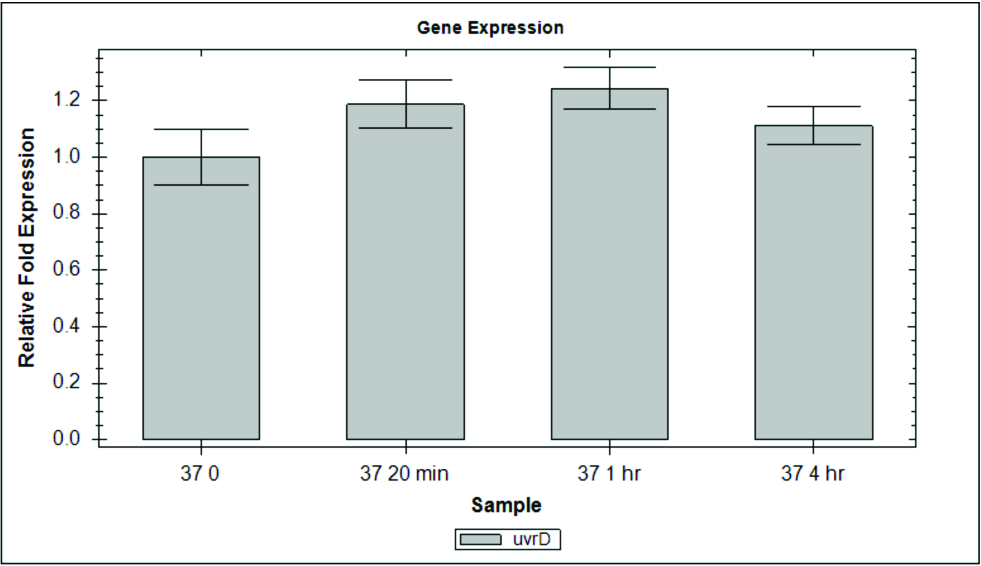

Supplement: Figure S1 — Expression of qRT-PCR reporter gene uvrD . Relative change in uvrD expression across multiple time points using RNA derived from U112 growth in TS broth supplemented with cysteine at 37°C. (TIF) [file pone.0019797.s001.tif]
